# Implementation of Patient Engagement Tools in Electronic Health Records to Enhance Patient-Centered Communication: Protocol for Feasibility Evaluation and Preliminary Results

**DOI:** 10.2196/30431

**Published:** 2021-08-26

**Authors:** Ming Tai-Seale, Rebecca Rosen, Bernice Ruo, Michael Hogarth, Christopher A Longhurst, Lina Lander, Amanda L Walker, Cheryl D Stults, Albert Chan, Kathleen Mazor, Lawrence Garber, Marlene Millen

**Affiliations:** 1 Department of Family Medicine University of California San Diego La Jolla, CA United States; 2 Department of Medicine University of California San Diego La Jolla, CA United States; 3 Division of Biomedical Informatics Department of Medicine University of California San Diego La Jolla, CA United States; 4 School of Medicine Department of Pediatrics University of California San Diego La Jolla, CA United States; 5 Palo Alto Medical Foundation Research Institute Palo Alto, CA United States; 6 Sutter Health Center for Health Systems Research Palo Alto, CA United States; 7 Sutter Health Clinical Leadership Team Sacramento, CA United States; 8 Stanford Center for Biomedical Informatics Research Stanford, CA United States; 9 Department of Medicine University of Massachusetts Medical School Worcester, MA United States; 10 Meyers Primary Care Institute University of Massachusetts Medical School, Reliant Medical Group, and Fallon Health Worcester, MA United States; 11 Reliant Medical Group Worcester, MA United States

**Keywords:** electronic health record, patient portal, patient–physician communication, agenda setting, patient priorities, patient engagement, health care teams, electronic health record documentation, standard work, digital health

## Abstract

**Background:**

Patient–physician communication during clinical encounters is essential to ensure quality of care. Many studies have attempted to improve patient–physician communication. Incorporating patient priorities into agenda setting and medical decision-making are fundamental to patient-centered communication. Efficient and scalable approaches are needed to empower patients to speak up and prepare physicians to respond. Leveraging electronic health records (EHRs) in engaging patients and health care teams has the potential to enhance the integration of patient priorities in clinical encounters. A systematic approach to eliciting and documenting patient priorities before encounters could facilitate effective communication in such encounters.

**Objective:**

In this paper, we report the design and implementation of a set of EHR tools built into clinical workflows for facilitating patient–physician joint agenda setting and the documentation of patient concerns in the EHRs for ambulatory encounters.

**Methods:**

We engaged health information technology leaders and users in three health care systems for developing and implementing a set of EHR tools. The goal of these tools is to standardize the elicitation of patient priorities by using a previsit “patient important issue” questionnaire distributed through the patient portal to the EHR. We built additional EHR documentation tools to facilitate patient–staff communication when the staff records the vital signs and the reason for the visit in the EHR while in the examination room, with a simple transmission method for physicians to incorporate patient concerns in EHR notes.

**Results:**

The study is ongoing. The anticipated completion date for survey data collection is November 2021. A total of 34,037 primary care patients from three health systems (n=26,441; n=5136; and n=2460 separately recruited from each system) used the previsit patient important issue questionnaire in 2020. The adoption of the digital previsit questionnaire during the COVID-19 pandemic was much higher in one health care system because it expanded the use of the questionnaire from physicians participating in trials to all primary care providers midway through the year. It also required the use of this previsit questionnaire for eCheck-ins, which are required for telehealth encounters. Physicians and staff suggested anecdotally that this questionnaire helped patient–clinician communication, particularly during the COVID-19 pandemic.

**Conclusions:**

EHR tools have the potential to facilitate the integration of patient priorities into agenda setting and documentation in real-world primary care practices. Early results suggest the feasibility and acceptability of such digital tools in three health systems. EHR tools can support patient engagement and clinicians’ work during in-person and telehealth visits. They could potentially exert a sustained influence on patient and clinician communication behaviors in contrast to prior ad hoc educational efforts targeting patients or clinicians.

**Trial Registration:**

ClinicalTrials.gov NCT03385512; https://clinicaltrials.gov/ct2/show/NCT03385512

**International Registered Report Identifier (IRRID):**

DERR1-10.2196/30431

## Introduction

Systematic reviews of the essentials for improving health care delivery have emphasized the importance of patient–physician communication [1,2]. Many efforts have been undertaken to improve patient–physician communication, including the use of a booklet to elicit patients’ agendas before visits and facilitating teach-back during visits [3], and implementing a systematic training program on patient-centered communication at the organizational level [4]. Efficient and scalable approaches are needed to empower patients to speak up and to prepare physicians to respond [3]. A component of an effective communication strategy is agenda setting, which aims to prioritize the items to discuss in a visit [4,5]. Agenda setting has been shown to improve patients’ health outcomes, satisfaction, and physicians’ time management [6]. However, it can be challenging for physicians to incorporate agenda setting in their workflow [7-10] even though doing so can facilitate visit management [11] without lengthening the visits [12].

The patient’s role in patient–physician communication is equally important. As electronic health records (EHRs) become an integral part of health care [13,14] patients often use the patient portal to communicate with their providers [15]. We created and implemented a set of EHR-based tools to facilitate joint agenda setting between patients and physicians and to ease the task of documenting patient priorities by physicians in the EHRs. We trained staff and physicians on the use of the tools as a part of an ongoing multicenter randomized control trial (ClinicalTrials.gov IHS-1608-35689). The aim of this paper is to report the design and implementation of our EHR tools into clinical workflow that facilitates patient–physician communication and present some preliminary interim results from the study.

## Methods

### EHR Tools

The research team collaborated with health information technology leaders at the University California San Diego Health (UCSDH), Sutter Health, and Reliant Medical Group to design, build, test, and implement the EHR tools for the study. All three health systems use a commercial EHR from the same vendor (Epic Systems). Representatives from across the three health systems attended weekly meetings to coordinate the patient-facing and care team–facing components during the EHR building and workflow implementation processes. The key components of the EHR built at UCSDH included the following: (1) a patient previsit questionnaire; (2) a new rooming tab for the staff; and (3) a documentation shortcut (SmartPhrase in Progress Notes) to enable physicians to see what the patient entered and incorporate it into the visit note. Considering organizational preferences, variations in the workflow existed across the three health systems. In addition to providing descriptions of these EHR tools, we present preliminary data on the feasibility of implementing these EHR tools in real-world primary care practices in diverse health systems located in Southern California, Northern California, and Central Massachusetts. Similar to other studies on the feasibility of Internet-based health interventions [13,14], we measured feasibility by seeking to understand participants’ acceptance of our tools, perception of the tools’ value in improving patient–clinician communication, and potential issues with their use. As the study is ongoing, the preliminary findings on the feasibility presented in this paper are informal. More formal analyses of user surveys involving clinicians, clinical staff, and patients will be performed after we complete data collection.

### Patient Previsit Questionnaire

**Figure 1:**
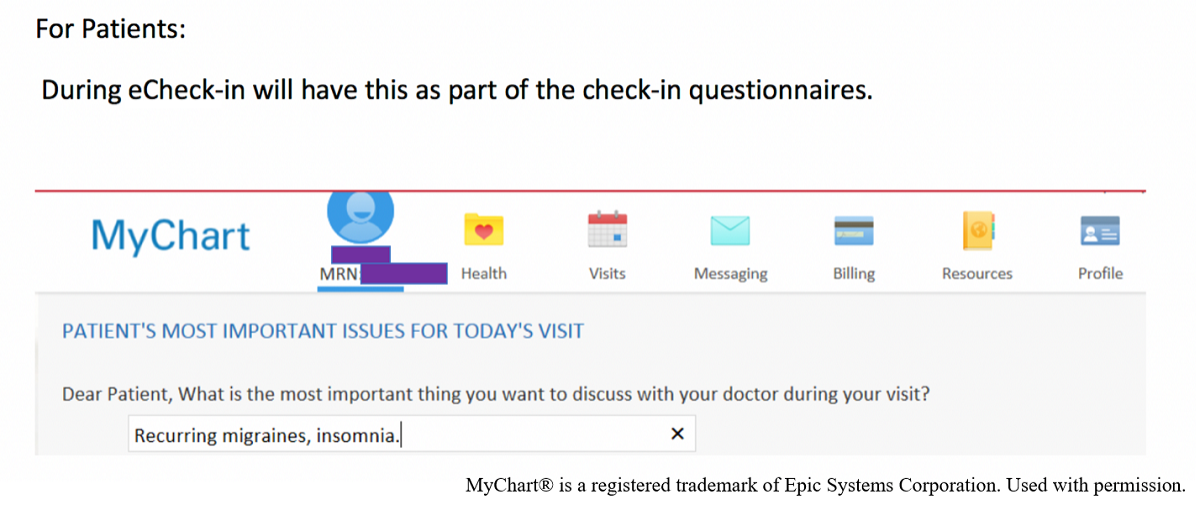
Previsit patient important issue questionnaire in MyChart.

The previsit questionnaire (Figure 1) was added as a check-in questionnaire in the EHR’s patient portal, known as “MyChart,” when a patient scheduled an appointment. The question, “What is the most important thing you want to discuss with your doctor during your visit?*”* appeared during the eCheck-in process. The text box was limited to 250 characters.

At Reliant, previsit patient questionnaires were attached automatically to appointments with primary care providers (PCPs) who were study participants when the visit was scheduled and released no sooner than 2 weeks before the visit date. Because legacy appointments could not automatically have the questionnaire, the Sutter and UCSDH teams manually attached it for the first 2 months of the study. As new appointments were scheduled, the questionnaire was automatically attached.

### Use of Patient Responses to the Previsit Questionnaire by Rooming Staff

Prior to meeting their physicians, patients are routinely greeted by clinic staff for visit preparation involving measuring vital signs and eliciting patients’ reasons for visiting. This process is commonly known as rooming. At UCSDH, once the patients arrived for their appointments, the rooming staff would see a new tab in rooming called “Patient Important Issues” in the EHR. This separate tab was created so that it would not be buried in the main rooming tab. The questionnaire also appeared before the EHR field called “Chief Complaint” so that it could be easily found. If the patients had already filled out the questionnaire, the staff could confirm the important issues with the patients. If the patients had changes or new issues to add, the staff could edit them accordingly. If the previsit questionnaire had not been completed, the staff could elicit and enter the patients’ responses.

No new tab was created at Reliant or Sutter. Early implementation at Reliant relied on the staff to copy and paste the patient’s response from the questionnaire into the “Chief Complaint” field and edit it as needed. Subsequently, the process was modified so that the patient’s response was automatically pulled into the rooming note if the staff used a documentation shortcut template for rooming. Use of this template was strongly encouraged but not required. Then, the staff could edit directly in the rooming note. At Sutter, the staff could see if the patient had completed the questionnaire in the visit schedule and proceed to view the response for that particular visit in the patient’s record. The staff verified the response with the patient and updated the record as needed.

### Integration of Patient Previsit Questionnaire Responses by Physicians

**Figure 2:**
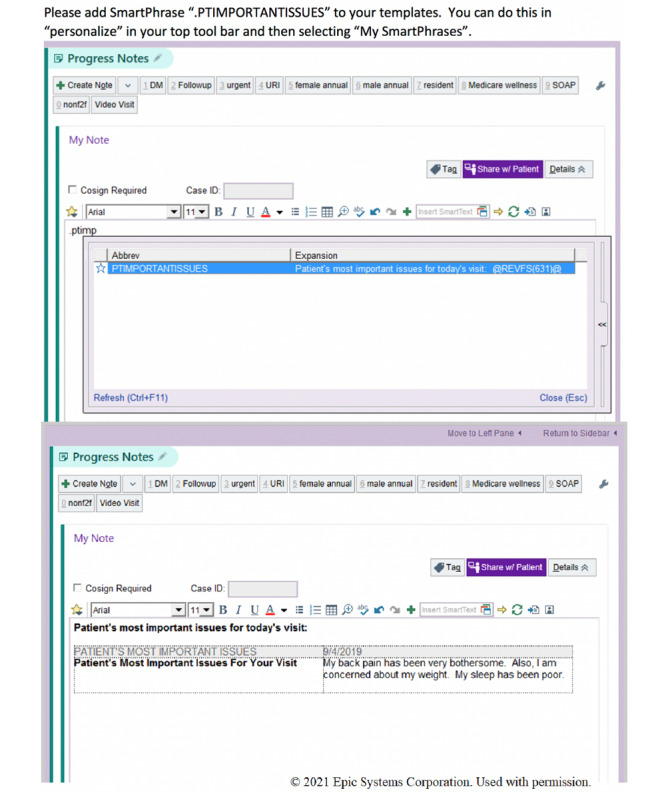
Documentation shortcut for physicians.

A documentation shortcut named “PTIMPORTANTISSUES” was created for physicians at UCSDH. Physicians were asked to add the shortcut to their documentation templates in the EHRs by personalizing their tool bar (Figure 2). Although it was also possible to add patients’ responses in notes during the appointment, physicians were encouraged to include the shortcut in their usual templates because it automatically added patients’ responses in their workflow, so they did not have to remember to look for them. After the patients were fully checked in, the physician could refresh the progress notes to see the patients’ most important reason (s) for their visit. This information is in a table format at UCSDH. Therefore, if the patient had responded to the questionnaire for previous visits, the physician could see a history of the issues by appointment date (Figure 2).

At Sutter, patient responses to the previsit questionnaire were incorporated into a shortcut “ENCQNR,” which physicians were encouraged to add to their documentation templates. A physician could designate the location in the progress notes by typing “ENCQNR,” and the previsit questions and patient responses would appear in a table format. The specific location of the patient response could vary based on the physician’s preference. As mentioned earlier, no new documentation shortcut was created specifically for the study at Reliant, but all the PCPs’ staff members were encouraged to use the shortcut for the rooming note template, which populated the patient’s responses to the questionnaire automatically at the top of the note.

### Interface With Telehealth During the COVID-19 Pandemic

Since the national declaration of the COVID-19 Pandemic in March 2020, all three systems rapidly increased clinical services delivered using telehealth [16]. The patients must state their important issues in the previsit questionnaire, as they are required for video visits in all the relevant clinics participating in the study at UCSDH. The UCSDH physicians have also added the documentation shortcut known as “SmartPhrase” to telehealth documentation templates. At Sutter, modifications were made to the existing previsit questionnaire to automatically attach the questionnaire for telehealth services with the participating PCPs. Furthermore, modifications were made to the physician’s documentation to include telehealth. At Reliant, the patient responses to the previsit questionnaire are available for telehealth services as they are for in-person visits.

## Results

### Survey Data Collection

The study is ongoing. The anticipated completion date for survey data collection is November 2021. We report interim findings on the adoption of patients’ most important issues into the EHR clinical workflow in the three health care systems.

**Figure 3:**
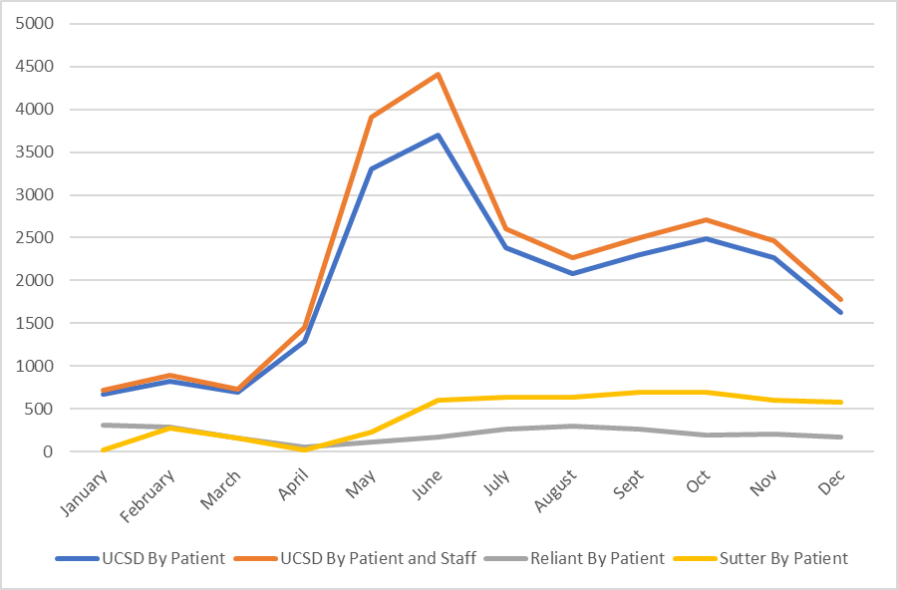
Responses to the previsit patient questionnaire across the three health systems in 2020. UCSD: University of California San Diego Health.

Between January 1 and December 31, 2020, the previsit questionnaire was used in 26,441 UCSDH visits, 5136 Sutter visits, and 2460 Reliant visits (Figure 3). At Sutter and Reliant, the previsit questionnaire was used only for the patients of the PCP participants involved in the study. UCSDH started with PCPs participating in the study and expanded it to all primary care visits at the request of the health system in June 2020. Because the questionnaire is required for eCheck-in before televisits, the increase in televisits at UCSDH during the surge of COVID-19 cases in the community could explain the pronounced upsurges in the use of the previsit patient questionnaire from April to July 2020.

### Feedback From Physicians and Staff

We have received informal comments and anecdotes from physicians. Three preliminary themes have emerged: acceptance of our tools; perceived values of the tools in improving patient–clinician communication, and potential issues with their use. Regarding acceptance of our tools, we heard comments such as “For the most part, patients have said this questionnaire was easy to use.”

The following comments reflect the perceived values of our tools in improving patient–physician communication:

There are no curve balls - I know what the patient wants to talk about before I see them.

For some of my more difficult/rambling patients, it may have provided a bit of focus.

It helps them focus and get more out of their medical visits.

I knew ahead of time what the issues were that we were going to discuss … have a form ready… made the appointment more efficient.

The patients listed something that they did not bring up so I was able to ask them about it.

Many patients are slightly anxious the first video visit so having a list to review made it easier to get started.

The staff also noted that the ease of incorporating patient responses into the rooming workflow empowered them to facilitate communication between the patients and health care team.

A few potential issues in using the tools were noted:

The biggest challenge is making sure the nurses are trained in pulling this question in. Some are familiar with the workflow and some are not, especially float nurses who are brought in as temporary help.

What I should have done was put it into my regular note but I kept losing track of the dot phrase. And this again tells me that I should fix this up to work better because it was helpful when it worked.

The staff reported that some patients entered health issues that they wanted addressed during their upcoming annual health exams. Although these visits are intended for preventive care rather than for addressing specific health issues [17], patients may view these as opportunities to discuss what matters to them with their primary care physicians. Physicians appreciate knowing these issues ahead of time as they can convert the annual health exams to office visits so that they are oriented to specific health issues that patients consider the most important during that visit. This helps promote patient-centered care. Identifying the patients’ important issues enables physicians to “take a step back and get their patients ready for the next time when they would undergo their annual health exam.”

## Discussion

Our study suggests that it is feasible to design and implement EHR tools to facilitate patient–physician communication, from joint agenda setting to documentation of patient priorities in physicians’ notes at the point of care. Consistent with the principles of translational informatics [18], our multilevel intervention nudges patients and clinicians with easy-to-use tools. It is in harmony with the “Meaningful Use” criteria, which emphasize patients playing an active role in their care via the patient portal [15]. The innovation of incorporating the patients’ most important issues into the EHR was intentionally designed for simplicity and ease of implementation. It has the potential to overcome the challenge of patient priorities not being integrated into the workflow [8]. Although each system has a different shortcut and method of using the previsit questionnaire, we show that this approach is feasible and can be implemented on a larger scale. Furthermore, for new digital tools and workflows, acceptance by end users is important. The acceptance level of our approach is illustrated by UCSDH’s application of the questionnaire more broadly to all primary care visits. Beyond our study, Epic has adopted this workflow on its platform and called it “Patient’s Most Important Issues” for all eCheck-ins to help identify patients’ reasons for scheduling an appointment.

We acknowledge that the central role of the patient portal in this effort may limit its reach to patients who do not use the patient portal either owing to limited access to the Internet or mobile phones. As a nation, digital infrastructure needs to be more accessible to all people regardless of where they live, and their health status, age, race, and education [19].

Our work demonstrates how a patient-centered communication intervention designed for a study, when implemented in alignment with health care delivery needs, can significantly benefit both endeavors. Furthermore, our approach is like OpenNotes [20], which enables patients, caregivers, and providers to jointly create clinical notes and care plans within the shared EHR. As more organizations adopt OpenNotes, where clinical notes are shared with patients [21,22], EHR interventions such as ours could empower patients to become more engaged in their health care.

## Abbreviations

EHRs: electronic health records
PCORI: Patient-Centered Outcomes Research Institute
PCPs: primary care providers
UCSDH: University California San Diego Health
